# Digital Health Tools for Managing Noncommunicable Diseases During and After the COVID-19 Pandemic: Perspectives of Patients and Caregivers

**DOI:** 10.2196/25652

**Published:** 2021-01-29

**Authors:** Alessandro Monaco, Katie Palmer, Nicolaj Holm Ravn Faber, Irene Kohler, Mitchell Silva, Anita Vatland, Joop van Griensven, Mariano Votta, Donna Walsh, Vincent Clay, Mehmet Cuneyt Yazicioglu, Danute Ducinskiene, Shaantanu Donde

**Affiliations:** 1 École des hautes études commerciales de Paris (HEC Paris) Jouy-en-Josas France; 2 Oliba Rome Italy; 3 The Danish Committee for Health Education Copenhagen Denmark; 4 Healthwatch Wiltshire Trowbridge United Kingdom; 5 Esperity Brussels Belgium; 6 Pårørendealliansen Oslo Norway; 7 Pain Alliance Europe Brussels Belgium; 8 Cittadinanzattiva/Active Citizenship Network Rome Italy; 9 European Federation of Neurological Associations Brussels Belgium; 10 Upjohn Brussels Belgium; 11 Upjohn Istanbul Turkey; 12 Upjohn Vilnius Lithuania; 13 Upjohn Surrey United Kingdom

**Keywords:** digital health, information and communication technologies, health technologies, telemedicine, noncommunicable diseases, COVID-19, SARS-CoV-2, patient advocacy, caregivers, ageing, patient empowerment, digital tool, perspective, patient perspective

## Abstract

**Background:**

A reduction in the number of face-to-face medical examinations conducted for patients with noncommunicable diseases (NCDs) during the first wave of the COVID-19 pandemic has led to health care professionals quickly adopting different strategies to communicate with and monitor their patients. Such strategies include the increased use of digital health tools. However, patient preferences, privacy concerns, a lack of regulations, overregulation, and insufficient evidence on the efficacy of digital health tools may have hampered the potential positive benefits of using such tools to manage NCDs.

**Objective:**

This viewpoint aims to discuss the views of an advisory board of patient and caregiver association members. Specifically, we aim to present this advisory board’s view on the role of digital health tools in managing patients with NCDs during and after the COVID-19 pandemic, and to identify future directions based on patients’ perspectives.

**Methods:**

As an initiative under the NCD Partnership (PARTners in Ncds Engage foR building Strategies to improve Healthy ageing In Patients) model of Upjohn, a web-based advisory board of patient and caregiver advocates was held on July 28, 2020, to bring together key stakeholders from public and private sectors.

**Results:**

The following key themes emerged: (1) technology developers should understand that the goals of patients may differ from those of health care professionals and other stakeholders; (2) patients, health care professionals, caregivers, and other end users need to be involved in the development of digital health tools at the earliest phase possible, to guarantee usability, efficacy, and adoption; (3) digital health tools must be better tailored to people with complex conditions, such as multimorbidity, older age, and cognitive or sensory impairment; and (4) some patients do not want or are unable to use digital health care tools, so adequate alternatives should always be available.

**Conclusions:**

There was consensus that public-private partnership models, such as the Upjohn NCD Partnership, can be effective models that foster innovation by integrating multiple perspectives (eg, patients’ perspectives) into the design, development, and implementation of digital and nondigital health tools, with the main overall objective of improving the life of patients with NCDs.

## Introduction

During the COVID-19 pandemic, there has been a justifiable focus on communicable disease prevention and management, which aims to control the risk of infection and transmission. However, noncommunicable diseases (NCDs) are still highly prevalent conditions [1,2], especially in older persons. NCDs accounted for an estimated 71% of worldwide deaths in 2016 [3,4].

During the first wave of the COVID-19 pandemic in 2020, many nonurgent outpatient services were closed to patients with NCDs; appointments were cancelled or postponed to reduce the risk of infection for patients and health care personnel and ease the burden on national health care services. These infection control procedures will have relevant implications for short- and long-term health, disease management, and quality of life of patients with NCDs [5]. This may be especially relevant to patients with complex conditions or multimorbidity (ie, the presence of 2 or more chronic conditions) [6,7]. Such patients usually benefit from integrated care, which includes comprehensive, coordinated care from multidisciplinary experts in multiple sectors [8-10].

Digital health tools include various technologies that can enhance health and health service delivery. Digital health tools can range from instruments that improve communication between health care professionals (HCPs) and patients, such as telemedicine technology, to electronic health records, mobile apps for monitoring symptoms, and medication and appointment reminder systems [11-16]. Digital health tools have been used for a range of purposes during the COVID-19 pandemic, including telemedicine service provision; remote patient monitoring; digital communication between political leaders and scientific authorities; and digital data monitoring for analyzing COVID-19 spread, COVID-19 evolution, and people’s perceptions [17,18]. However, most countries lack a regulatory framework to authorize, integrate, and reimburse telemedicine services, and there is large heterogeneity between countries [19].

Even before the COVID-19 pandemic, many have emphasized that digital health tools are important for the prevention and management of NCDs [20], as digital health tools play an essential role in integrated care [9,21], especially in managing multimorbidity [8-10,22], delivering precision medicine [23], and increasing medication adherence [24]. However, older patients are less likely to choose telemedicine visits over office visits than younger patients, and other factors, such as access to technology, also play a role in patients’ preferences for service delivery modes [25]. Patients’ engagement with digital health tools is affected by multiple factors, including individual motivations, personal life values, approaches for facilitating engagement and recruitment, and the quality of the tool [11].

Since the start of the pandemic, there has been a rapid surge in the use of telemedicine tools and digital health tools for addressing non–COVID-19 medical issues [26]. However, patient preferences, privacy concerns, and insufficient evidence on the efficacy of digital health tools may hamper the potential positive benefits of such tools in the management of NCDs. Since the start of the pandemic, research on the use of digital health tools has mostly focused on how these instruments can be used to screen and diagnose COVID-19 [18]. This raises important questions about how these tools will be used in the health care of patients with NCDs in the postpandemic era. Although evidence-based results on clinical efficacy, ease of use, and cost-effectiveness from the perspective of HCPs, health care institutions, and national health services are important, we must also consider the needs, abilities, and wishes of the patients and caregivers who will ultimately be using and benefiting from these tools. We need to know whether patients want to manage their NCDs with digital health tools in the future, and understand the aspects that should be considered as we move forward to the next stage of the pandemic.

The objective of this viewpoint is to present the perspective of patients with NCDs and caregivers, with regard to the use of digital health tools for the prevention and management of NCDs. This viewpoint does not present a scientific summary of published evidence. Instead, it focuses on the aspects that patients and caregivers deem important for NCD prevention and care, in the context of an advisory board. Our specific aims include (1) describing the evolving scope of digital health tools for NCDs in the advent of the COVID-19 pandemic, and discussing patients’ opinions on how these tools can be better designed and developed; (2) outlining ways to increase patient and caregiver engagement to optimize digital health tools; (3) presenting the challenges that patient and caregiver organizations perceive to be barriers to the use of digital health tools; and (4) identifying research priorities for improving the future use of digital health tools in NCD management.

## Methods

The content of this viewpoint summarizes the consensus discussion of an advisory board within the NCD Partnership (PARTners in Ncds Engage foR building Strategies to improve Healthy ageing In Patients), which is an initiative that brings together different stakeholders from public and private sectors, so that they can work together to find tools for increasing healthy aging and reducing the burden of NCDs. This initiative involves an institutional collaboration between Upjohn, a division of Pfizer, and the European Innovation Partnership on Active and Healthy Ageing. The NCD Partnership has been engaged in numerous activities, including the creation of several advisory boards that consist of policy makers, physicians, HCPs, researchers, patient and caregiver advocacy groups, patient empowerment organizations, and industry experts.

On July 28, 2020, Upjohn and the European Innovation Partnership on Active and Healthy Ageing organized a web-based advisory board that consisted of patient and caregiver advocacy group members and patient empowerment organizations. The EUPATI (European Patients’ Academy on Therapeutic Innovation) Guidance for Patient Involvement in Medicines Research and Development: Health Technology Assessment [27] has suggested that consensus building excercises should be one of the patient involvement activities.

The NCD Partnership has been operating in the field of NCDs and healthy aging for more than 2 years, since 2018. Several advisory boards that consist of researchers, HCPs, and patient advocacy groups have already been created by the NCD Partnership (ie, in September 2018, September 2019, June 2020). Furthermore, the partners of the NCD Partnership have published several papers based on information that was provided in meetings. These papers formed the basis of the advisory board in this viewpoint.

The organizing committee consisted of an expert (AM) in personalized medicine, health care administration, and regulatory affairs; an epidemiologist (KP) with extensive research expertise in NCDs, multimorbidity, and aging disorders; and a physician (SD) with expertise in pharmaceutical medicine, digital initiative leadership, and partnerships for improving patient outcomes. Both AM and KP have extensive experience in the public sector with regard to chairing and conducting consensus meetings, preparing clinical guidelines, and working with researchers, HCPs, and advocacy groups.

In addition to the chairman (AM) and epidemiologist (KP) who led the consensus dialogue, the advisory board included 7 members from patient and caregiver organizations who were recognized by the organizing committee. We aimed to include a wide range of organizations with views on the different aspects of NCDs including caregiver involvement; patient empowerment; health literacy and patient education; patient rights; and specific priority NCD themes, such as aging, pain, and neurology.

In terms of this viewpoint’s methodology, we first identified several themes from previous NCD Partnership advisory board meetings that reflected major barriers to NCD management for patients, including integrated care [21], health technology [20], and COVID-19–related changes to the management of risk factors and diseases in patients with NCDs [5]. Afterward, we prepared a draft of talking points based on these themes. This draft was sent to the members of the advisory board. Each member was given time to discuss with their organizations and obtain feedback from patients, caregivers, and other experts from their associations. We then organized individual web-based meetings with the organizing committee and each advisory board member. During these meetings, the advisory board member provided feedback on the talking points. They were asked to add, remove, or modify the discussion points based on comments from their organization, and identify priority areas for discussion. A new set of talking points was then drafted and sent out to advisory board participants before the final meeting. The final meeting was a 1-day, web-based event that lasted for 7 hours. This meeting included several scheduled breaks throughout the day to ensure that any advisory board members with health issues had ample time to rest or move around. AM acted as the chairperson, while the epidemiologist (KP) documented the views, disagreements, and areas of consensus. A qualitative discussion was conducted, and this continued until a consensus was reached. In case a consensus was not reached, a list of areas for future research was made, as reported in the Results section. The meeting was also recorded to allow the chairperson and epidemiologist to review all discussions and identify key themes and recommendations.

## Results

### Patients’ and Caregivers’ Perspectives of Digital Health Tools in NCD Management

#### Summary of Results

The results section of this viewpoint provides a summary of the consensus points that were agreed upon by the patient and caregiver organizations. These consensus points were related to the use of digital health tools for preventing and managing NCDs. We provide several patient and caregiver perspectives and present their opinions on how digital tools can be optimized during and after the COVID-19 pandemic to improve patient outcomes. The results section reflects the perspectives of the patients and caregivers that the advisory board participants represent.

#### Consensus Theme 1: The Evolving Scope of Digital Health Tools for NCDs in the Advent of the COVID-19 Pandemic, and Patient and Caregiver Perspectives on How These Tools Can Be Better Designed and Developed

The COVID-19 pandemic has prompted a rapid change in the use of digital health tools. The advisory board members felt that the pandemic has provided the opportunity for scaling up the development of these tools so that they can be used to manage both communicable diseases and NCDs. During the pandemic, there was a rise in the use of digital tools for increasing communication between HCPs and patients (eg, telemedicine and video consultation technologies). Digital health tools can be used as an alternative when nonurgent face-to-face consultations are cancelled. Furthermore, these tools can be used for COVID-19 symptom tracking and tracing. These changes may have a positive and negative impact on the future use of digital health tools in health care. During the consensus discussion, the patient and caregiver advocates felt that the pandemic may have triggered the opportunity for citizens to engage in the use of digital tools and participate in studies that collect data on health and NCD prevention. However, some people may be concerned about ethical and privacy issues. They may also be skeptical about replacing face-to-face health care with digital alternatives. There was consensus that digital health development should be part of an overall long-term strategy that advances in parallel with the greater investments that multiple clinical and nonclinical settings have put into medicine and integrated care. The COVID-19 pandemic is likely to result in the reconfiguration of care pathways in the short- and long-term future, and this reconfiguration may lead to a considerable increase in community- and home-based care, wherein digital tools can support patient management. In the following section, we discuss what roles patients and caregivers can play in the design and development of digital health tools, and whether tools used during the COVID-19 pandemic can play a part in the future management of NCDs.

The advisory board members proposed that a comprehensive range of digital health tools that take into account the whole life of an individual (ie, including the epigenetic life cycle) should be used. This includes an individual’s preconception, birth, childhood, adulthood, and older age. These tools should also provide methods for primary prevention (eg, control of lifestyle and treatment of risk factors in healthy individuals), secondary prevention, tertiary prevention, and management of NCDs (eg, symptom management for people in a disease state). To prevent NCDs, digital health tools can include exercise monitors, fitness watches, sleep monitors, and apps that monitor lifestyles and NCD risk factors. Tools that focus on disease and symptom management can include a variety of individual-level instruments (eg, medication reminder systems, adherence monitoring systems, etc), organization-level instruments (eg, appointment scheduling systems, electronic health records, etc), or instruments that improve communication between HCPs and patients (eg, teleconsultation technologies, remote symptom monitoring technologies for blood pressure control, wearable glucose monitors, etc). Different individuals who are involved in the care process may be the target users of digital health tools. Such individuals include community nurses, pharmacists, and people who play an important role in patients’ care outside of clinical settings [28]. The perspectives of patients and caregivers may differ depending on the goal of the digital health tool and the type of technology used. The advisory board members felt that it was important to identify the target users for each type of technology and have clear objectives for each tool. The profile of patients with NCDs can differ dramatically in terms of their characteristics and the type of disease or diseases that they have. For example, an oncological patient might have very different needs compared to a person with a neurological disease.

There was strong consensus that digital health tools should be used to enhance health care, rather than transition from nondigital NCD management to fully digital NCD management. Although HCPs and patients with NCDs do not usually have the option to manage NCDs through face-to-face consultations during pandemic-related lockdowns and periods of shielding or quarantine, it is important to identify which digital health tools provide the best solutions for patients and HCPs, and ensure that such tools do not replace better alternatives in nonpandemic times. Appropriate digital health tools might differ depending on situational changes, and people need to have a clear motivation for using digital tools instead of nondigital tools. For example, during the COVID-19 pandemic, patients have clearly benefited from teleconsultations because they minimize the contact between patients and HCPs and reduce the risk of contracting SARS-CoV-2. However, many of the advisory board members emphasized that some individuals want face-to-face contact with HCPs once social distancing measures have eased because they miss human contact. This may be relevant to specific patient groups who prefer nondigital contact with HCPs. On the other hand, some patients, such as those with certain psychiatric disorders, may prefer to receive remote medical care in a home setting.

#### Consensus Theme 2: Methods for Increasing Patient and Caregiver Engagement to Optimize Digital Health Tools

The advisory board members agreed that digital health tools need to demonstrate a clear benefit for patients, to increase their engagement with these tools. If individuals cannot perceive the personal advantages of using a specific tool, it is less likely that they will use it. On the other hand, institutions have analytic tools that can assess the cost-effectiveness of adopting innovative tools and predict the socioeconomic impact of technological solutions. The patient and caregiver advocates emphasized that outcomes need to be meaningful to the patient and take into account individual aspirations. Further, there needs to be clear definitions for what a digital health solution aims to do. For example, a tool can be designed to improve the frequency of communication between HCPs and patients, prevent disease onset, improve adherence to therapies, manage multimorbidity, or help caregivers. During the COVID-19 pandemic, the purpose of digital health tools has mainly been to increase communication between patients and HCPs. However, there are many different goals that can be achieved via digital health tools (eg, symptom monitoring, health tracking via biosensors, risk factor control, and medication adherence), and some of these goals may be important to other end users, such as caregivers, HCPs, or health care systems. Therefore, digital health tool developers should understand that the goals of patients may differ from those of HCPs and other stakeholders. Outcomes that may be deemed more relevant from an HCP perspective (eg, controlling symptoms, increasing medication adherence, etc) might not be as relevant from a patient perspective, in terms of quality of life and other aspects (eg, patients are often more interested in concrete goals, such as visiting grandchildren, being able to go to a concert, gardening, etc). If the HCP perspective is not in line with the end user perspective, any developed digital health tools are likely to be unsuccessful.

The advisory board members felt that HCPs, caregivers, and other end users need to be involved with the development of digital health tools at the earliest phase possible, to increase the chances of developing technologies that people are motivated to use. These technologies should also have a high impact on patient outcomes. Ideally, the involvement of end users should occur before the implementation phase. Carefully designing and developing digital health tools will hopefully improve the life of patients and caregivers by helping them achieve meaningful goals that have an impact on their quality of life. However, even if better-designed tools become available, this does not guarantee that they will be widely used.

Patients’ engagement with digital health tools is affected by multiple factors, including individual motivations and personal life values. However, the advisory board members stressed that during the COVID-19 pandemic, many patients with NCDs have not had many viable alternatives to routine medical care; patients have no choice but to use telemedicine tools. Although telemedicine techniques are essential for helping HCPs maintain contact with patients with NCDs during periods of social distancing and lockdown, there should be a clear discussion and assessment of which digital health tools will be best used in the future management of patients with NCDs, and whether such tools are able to adequately replace or enhance nondigital health care techniques. Research, product development, and training must focus on individuals who will ultimately benefit from digital health tools as well as methods for improving tool usability (ie, improving user interfaces to facilitate adoption).

There was an overall consensus for improving potential users’ engagement with digital health tool by developing instruments that focus on the positive aspects of a tool, instead of just the negative aspects (eg, using language such as “prevention and health” rather than “disease and symptoms”). Technologies that monitor changes in symptoms may not provide impactful incentives that adequately engage patients. Such technologies even run the risk of demotivating individuals during periods of NCD progression and symptom deterioration. Thus, digital health tools should have distinct purposes based on target users and their goals. For example, sports watches should be promoted as preventative tools for healthy individuals who want to monitor their fitness and reduce the risk of developing a future NCD, whereas people who already have NCDs might become distressed from receiving continuous feedback on their health data. Therefore, people with NCDs might better engage with tools that focus on more concrete changes that are relevant to their needs. Patients with NCDs are often motivated by specific and personalized goals, such as being able to independently manage their everyday life.

The advisory board members suggested that it is challenging to have individuals engage with digital health tools over long periods of time. People are often more motivated to use health-related technology when they receive a new diagnosis or experience symptoms. However, when their symptoms improve, the urge to use digital health tools can decrease. Thus, in addition to increasing new users’ engagement with digital health tools, it is important to find methods for increasing motivation in people who have recovered or have not experienced severe disease symptoms, to prevent reoccurrence.

Increasing patients’ engagement with digital health tools can also be achieved by designing or adapting digital health tools to be more user-friendly, and by focusing on the specific limitations (ie, physical, functional, and cognitive limitations) that patients with NCDs may experience. It is imperative to develop strategies that limit the time and effort needed to input data, so that simpler, more time-efficient tools can be created. It is also essential to tailor digital health tools to individuals with limited experience of technology (eg, older persons), and to adapt technologies so that they can be better used by people with specific needs, such as those with cognitive or sensory impairment.

Many people with NCDs, especially those with multimorbidity, are older individuals. The advisory board felt that age is an important factor to consider, because older individuals may be less likely to use digital health tools than younger people, and specific strategies are needed to engage older individuals. For example, isolated older persons who live in remote areas might not have access to technology or may not use the internet. The patient and caregiver advocates agreed that digital health approaches must be sensitive to the needs and wants of older people. Furthermore, the advisory board members questioned whether HCPs should continue to have patients engage with nondigital methods, or try to change the situation by providing patients with tools, technology, or training for using new digital health care tools. There will always be a proportion of the population who will not want or will not be able to use a digital health care solution. Therefore, there should always be adequate alternatives available to those who want them.

#### Consensus Theme 3: Challenges That Patient and Caregiver Organizations Perceive to Be Barriers to the Use of Digital Health Tools

The advisory board members discussed how to best use digital health tools in the prevention and management of NCDs in the short- and long-term future. When developing innovative solutions, there need to be clear outcomes and benefits for all patients and stakeholders. There was concern that the rapid development of digital health tools might not necessarily result in the best care of patients. Furthermore, whether patients and HCPs like using digital health tools, and whether they prefer digital health tools over nondigital alternatives still needs to be established. Patient advocacy organizations can help to improve knowledge by investigating patients’ motivations for using digital health tools. For example, Pain Alliance Europe [29] conducted a web-based survey in 2020 to collect information on the use of eHealth (ie, the use of information and communication technologies for health purposes) and mobile health (mHealth; ie, the use of mobile devices for medical and public health practices) services. Data were collected from 1789 patients with chronic pain from 28 European countries. The responses showed that 46.28% (827/1787) of individuals did not use eHealth or mHealth services. The use of such services varied between different countries and age groups; the frequency of a lack of use was generally higher in older age groups (ie, >50 years) than in younger age groups. One of the main reasons that patients did not use eHealth or mHealth services was because they did not know about them (422/779, 54.17%). Additionally, 16.17% (126/779) of participants did not use such services because they did not see how these services would benefit them as an individual. The most common reason for using eHealth and mHealth services was because patients could use them to self-manage their conditions. With regard to the patients who did use eHealth or mHealth services, almost half (317/472, 48.84%) did not share their health data with anyone, while 9.86% (64/472) of patients shared data with their primary care physician. Future surveys that evaluate patient preferences, motivations, and patterns of use can help identify barriers to the use of digital health tools and different patterns that are based on the types of NCDs and symptoms.

Another major concern of the advisory board members was whether the use of digital health tools in health care will lead to inequalities among patients. Nondigital alternatives are needed for people who are unwilling or unable to use digital health technologies, and specific groups (eg, older persons, migrants, and people with cognitive or sensory impairment) should not be disadvantaged if they do not use digital health tools. Inequalities among patients may be relevant to different aspects of digital health tool use, including access to technology, training, digital health literacy, and motivation to use them. Many individuals have expressed concerns about data privacy and who their information will be shared with (eg, insurance companies, banks, or employers). It is unlikely that patients will fully embrace digital health care tools until privacy concerns are adequately addressed.

#### Consensus Theme 4: Research Priorities for Improving the Use of Digital Health Tools in Future NCD Management

The advisory board members agreed that before digital health tools are used on a wider scale, high-quality research is needed to investigate how effective they are in the prevention and management of NCDs during the COVID-19 pandemic era and beyond. The patient and caregiver advocates devised several concrete research questions that should be prioritized. The questions about the individual patients’ characteristics, needs, and wants, and the questions about how digital health tools will improve patient outcomes and quality of life are particularly important. Specific research objectives are proposed in Textbox 1. The advisory board members were also concerned about the amount of current evidence that is based on clinical outcomes that have been defined and assessed by HCPs, as there is often a lack of patient and caregiver perspectives in the literature. Research should clarify the distinctions between digital health tools that are developed for healthy people (eg, digital health tools that help prevent disease in the long term) and those developed for patients (eg, digital health tools that improve patient outcomes via symptom monitoring or increasing drug adherence). A key topic for research is determining whether motivations for using digital health tools differ among patients with different diseases or characteristics. For example, if the goal is to increase physical activity levels, digital health tool developers need to know which people are driven by face-to-face interventions and which people prefer using wearable devices. We also need to build registries to identify biomarkers and risk factors for so-called “unpreventable diseases.” Digital health tools may help with gathering and analyzing data on a wider scale. It is also vital to develop and design health technologies while carefully considering the European Charter of Patients’ Rights [30].

**Research questions on digital health tools in noncommunicable disease management, as suggested by patient and caregiver organizations on the advisory board.**

**Research questions**

- How did patients and health care professionals view the use of telemedicine during the first wave of the COVID-19 pandemic? Did these tools have a positive impact on patient management and symptom control in the short and long term?
- How do patients and health care professionals feel about teleconsultations? Do patient outcomes differ depending on whether care is provided through remote telemedicine tools versus face-to-face methods? Are there specific diseases that these solutions work better for (eg, people with social anxiety might prefer teleconsultations)?
- Which type of digital health tools have a greater impact on the outcomes of individuals and is it always a positive impact?
- Do patients and health care professionals want to continue using telemedicine and other digital health tools after the COVID-19 pandemic, and if so, which ones?
- Which demographic groups are more likely to use digital health tools and how can the other groups be encouraged to use tools more frequently?
- How effective is technology in increasing behavioral change? Do changes in behavior affect clinical outcomes or help patients achieve meaningful goals?
- Which clinical changes have an effect on a patient’s quality of life and how can health care professionals better assess what patients’ individualized goals are?
- Is quality of life improved by better medication adherence or do changes such as symptom control or psychological support have a bigger effect? Do these differ based on disease, age, gender, and other characteristics?
- Can single digital health tools be adapted to the specific goals of individual patients?
- What motivates individual people to use digital health tools? Does motivation differ based on disease type and other patient characteristics?

## Discussion

In conclusion, the COVID-19 pandemic has instigated a rapid increase in the use of digital health tools, and has highlighted the need for developing better tools that are adequately suited to the future needs of both HCPs and patients. It is still unclear how the COVID-19 pandemic will develop; it seems likely that we will face new outbreaks and repeated lockdowns that will result in a renewed disruption of NCD health services and management. Digital health tools can help with providing alternatives for nonurgent, face-to-face medical and care services during lockdown periods. However, digital health tools can also be used to further enhance NCD prevention and management by integrating them into health care services during nonpandemic times. Furthermore, patients’ and caregivers’ opinions are essential for driving the development of effective tools with the maximum potential impact for end users. Patient and caregiver advocacy groups, citizen networks, and empowerment organizations need to be better integrated into all phases of digital health tool development. This will ensure that meaningful patient and public involvement occurs. Concrete goals need to be set for increasing patients’, caregivers’, and HCPs’ digital literacy. We also need to build and enhance well-connected infrastructures that allow for equitable access to digital health tools. Current regulatory and reimbursement frameworks also need to be reviewed so that they can be adapted to the changing health care pathways that are likely to be increasingly supported by future digital health tools.

We need to clarify how to facilitate patient and caregiver association involvement in the research, development, design, and innovation of care processes and digital health care tools. Our viewpoint highlights the importance of public-private partnership models (eg, the NCD Partnership) and other initiatives (eg, the EUPATI [27], the Partners Forum [31], and the NCD Alliance [32]) in fostering innovation to achieve meaningful goals. The NCD Partnership is an initiative that aims to create a platform for debate and facilitate the collaboration of policy makers (eg, the European Commission), HCPs, industry experts, researchers, patients, and caregivers, by creating advisory boards, implementing collaborative research projects, and conducting other activities. It has been emphasized that successful plans for addressing NCDs require international-level, national-level, and local-level partnerships among national governments, private sectors, and civil societies, and the involvement of a wide range of key actors, including patients and their families [33]. Other multistakeholder partnerships, such as the EUPATI, have emphasized the importance of patient involvement in health technology assessment [27], and have suggested that consensus-building exercises can be used to improve patient involvement. Strengthening the collaboration between public and private organizations that have a strong involvement in civic and patient associations (ie, in terms of health literacy and digital health literacy) can help increase people’s ability to obtain, read, understand, and use information that relates to digital technologies. By bringing together multiple stakeholders, including patients, into the design, development, and implementation of digital health tools, we can make advancements that will help align the goals of all partners who share an overall objective of improving the life of patients with NCDs.

## Abbreviations

EUPATI: European Patients’ Academy on Therapeutic Innovation
HCP: health care professional
mHealth: mobile health
NCD: noncommunicable diseases
NCD Partnership: PARTners in Ncds Engage foR building Strategies to improve Healthy ageing In Patients
